# Reporting on patient and public involvement (PPI) in research publications: using the GRIPP2 checklists with lay co-researchers

**DOI:** 10.1186/s40900-021-00295-w

**Published:** 2021-07-22

**Authors:** Julia Jones, Marion Cowe, Sue Marks, Tony McAllister, Alex Mendoza, Carole Ponniah, Helena Wythe, Elspeth Mathie

**Affiliations:** 1 Centre for Research in Public Health and Community Care (CRIPACC), University of Hertfordshire, Hatfield, AL10 9AB England; 2 Public Involvement in Research group (PIRg) member, CRIPACC, University of Hertfordshire, Hatfield, AL10 9AB England; 3 NIHR ARC East of England, School of Health Sciences, University of East Anglia, Norwich, NR4 7TJ England

**Keywords:** Patient and public involvement, PPI, Co-researchers, Patient and public peer review, GRIPP2 checklists

## Abstract

**Background:**

Patient and public involvement (PPI) in health and social care research is considered important internationally, with increasing evidence that PPI improves the quality, relevance and outcomes of research. There has been a growth in research publications that describe PPI in the research process, but the frequency and detail of PPI reporting varies considerably. This paper reports on a collaborative study that aimed to describe the extent of PPI in publications from research funded by the Collaboration for Leadership in Applied Health Research and Care (CLAHRC) in the East of England (EoE), part of the National Institute of Health Research (NIHR) in England (2014–2019).

**Methods:**

A descriptive study of all research publications (1st January 2014 to 31st October 2017) funded by the NIHR CLAHRC EoE. Members of the Public Involvement in Research group (PIRg), at the University of Hertfordshire, were actively involved, with four PIRg co-researchers. We used an internationally recognised reporting checklist for PPI called the GRIPP2 (Guidance for Reporting Involvement of Patients and the Public, Version 2) to guide the reviewing process.

**Results:**

Out of 148 research papers identified, 16 (14%) reported some aspect of PPI activity and were included for review. Ten of the publications (63%) acknowledged the contributions of PPI individuals and/or groups and five had PPI co-authors. There was considerable variation in the PPI reported in the publications, with some ‘missed opportunities’ to provide detail of PPI undertaken. The perspectives of the co-researchers shaped the reporting of the results from this study. The co-researchers found the GRIPP2-SF (short form) to be useful, but the GRIPP2-LF (long form) was considered over complicated and not user-friendly.

**Conclusions:**

This is one of the first studies to involve lay co-researchers in the review of PPI reporting using the GRIPP2 reporting checklists (GRIPP2-SF and GRIPP2-LF). We make recommendations for a revised version of the GRIPP2-SF, with clearer instructions and three additional sections to record whether PPI is reported in the abstract or key words, in the acknowledgements section, and whether there are PPI co-authors. We also recommend the provision of training and support for patient and public peer reviewers.

**Supplementary Information:**

The online version contains supplementary material available at 10.1186/s40900-021-00295-w.

## Plain English Summary

Involving patients, family carers and members of the public in research is known as patient and public involvement, or PPI. In health and social care research, PPI is considered important by many, including patients and research funders. Increasingly, research publications include an account of how members of the public have been involved in research being reported. But often there is not much detail regarding who was involved, what they did, and what difference their involvement made.

In our study, we reviewed publications from research funded by an organisation called the Collaboration for Leadership in Applied Health Research and Care (CLAHRC) in the East of England (EoE), to understand how members of the public had been involved. We also wanted to see how lay co-researchers found the use of a checklist developed to assess the reporting of PPI: the ‘Guidance for Reporting Involvement of Patients and the Public, Version 2’ (GRIPP2).

Members of a Public Involvement in Research group (PIRg), based at the University of Hertfordshire, were actively involved in the study, with four PIRg members becoming co-researchers. The GRIPP2 has a long form and short form, and we used these to review 16 research publications.

We found great variation in the PPI activities reported in the publications. The lay co-researchers thought the GRIPP2 short form was useful, but the long form was considered over complicated and not user-friendly. We recommend a revised version of the GRIPP2 short form, with clearer instructions, additional sections and training and support for patient and public reviewers.

**Supplementary Information:**

The online version contains supplementary material available at 10.1186/s40900-021-00295-w.

## Background

Internationally, the importance of involving patients, carers and members of the public in the design and conduct of health and social care research is well established [1–3]. Evidence that public involvement can improve the quality, relevance and outcomes of research is growing [4, 5] with an acknowledgement that patient and public involvement (PPI) can play an important role in reducing health and social care research waste [6]. There are also strong ethical and political arguments for public involvement in decision-making about health and social care services and research, as a right of citizens [7, 8]. The term patient and public involvement (PPI) is widely used to describe research that is being carried out ‘with’ or ‘by’ members of the public, rather than ‘to’, ‘about’ or ‘for’ them”. Members of the public include a wide range of people including: patients, potential patients, family carers, service users, people using health and social care services as well as people from organisations that represent people who use services [9]. To support this growing area of research, a range of patient and public involvement (PPI) guidelines, frameworks and standards have been developed for researchers and PPI contributors alike [9–13]. In the UK, National Standards for Public Involvement were launched in 2018 and updated in November 2019 to “improve the quality and consistency of public involvement in research” [14] .

In recent years, there has been a rise in the number of research articles that report PPI in health and social care research. The reporting of PPI has been observed to range from a brief comment, to a detailed discussion of how PPI contributors were actively involved in the research [5, 15, 16]. The quality of the PPI reporting varies greatly, from the detail of the involvement described, whether PPI contributors and researchers reflect on the process of involvement, and any acknowledgment of named individuals and/or patient groups or organisations [17, 18]. As described by Bowers et al. [19], when PPI is mentioned in publications, the reporting is rarely specific about the input of public contributors and the PPI often remains ‘a black box’ (p220). This reduces the opportunity for shared learning about what works for whom, in what circumstances and why [20, 21].

A scoping exercise of PPI in research in the UK [22], published in 2014 as part of the RAPPORT study [20], found that 51% of the182 research studies included in the scoping exercise reported some element of PPI in the research conducted. This information was collated from a number of different sources, including email exchanges with researchers, published and unpublished project documents. The scoping review found limited information about the extent of PPI reported in the publicly accessible documents from these studies, such as research protocols, reports, journal articles and project websites. More recent reviews of the quality and frequency of reporting of PPI have been conducted on topics including dementia [2] and surgical research [23], across different study designs [24], clinical trials [25] and within an individual medical journal [17]. A study of the frequency of PPI reporting in the British Medical Journal (BMJ) by Price and colleagues [17] demonstrated the impact of a new journal policy for authors to report PPI in their papers [26], which led to an increase from 0.5% (2013/14) to 11% (2015/16) of PPI reporting in the 12 month post-implementation period. However, despite an increase in the frequency of reporting, the authors concluded that the quantity and quality of reporting remained low and inconsistent. This study also revealed that PPI contributors were often not fully acknowledged for their involvement, and only 12% of the papers reviewed had PPI co-authors. A systematic review of patient engagement in clinical trials by Fergusson and colleagues [25] found less than 1% of the 2777 clinical trials reviewed had reported any meaningful engagement of patients. The authors described the engagement of patients in intervention research as ‘very poor’. However, some authors question the focus on measuring the frequency and impact of PPI as a research outcome, particularly in quantitative terms, and call for a re-conceptualisation of public involvement as conversations and learning between researchers and the public [21, 27].

Over the last decade, research by Sophie Staniszewska and colleagues [5, 18, 28] has highlighted the importance of clear guidance to improve the reporting of PPI in research. The first version of the Guidance for Reporting Involvement of Patients and the Public (GRIPP), published in 2011 [28], was based on findings from systematic reviews [5, 29] with the aim of improving the quality, transparency and consistency of PPI reporting. Version 2 of this guidance, published in 2017 and called GRIPP2, was subsequently developed with PPI contributors to be more focused on what patients and the public think is important to report. The GRIPP2 has two versions, a Long Form (LF) and Short Form (SF). The Long Form (GRIPP2-LF) was designed for studies where the main focus is PPI, with the short form (GRIPP2-SF) aimed at other types of studies [30].

It is interesting to note that some previous reviews on the reporting of PPI have involved members of the public and also utilised the GRIPP and GRIPP2 forms to structure the review process. The review of PPI reporting within surgical research by Jones and colleagues [23], (published pre-GRIPP2), used the original GRIPP checklist, in combination with a critical appraisal tool for user involvement in research [31] to describe the reporting of PPI in eight articles. A patient co-researcher was involved in all stages of the review and was named as a co-author. The review by Miah and colleagues [2], published in 2019, involved three members of a dementia focused research advisory group in the interpretation of the review findings. This review utilised the GRIPP2-SF to describe and summarise the data, but this process was conducted by the research team, not the members of the advisory group. Interestingly, the authors adapted the GRIPP2-SF by adding some additional fields, including: the country where the research was conducted; who was involved (patients and public); the PPI terms used; and methods used to evaluate the impact of the PPI.

In our study, we have used the GRIPP2 forms (Long and Short) as a framework to guide our description of different types of research publications that reported research funded by the NIHR Collaboration for Leadership in Applied Health Research and Care (CLAHRC) in the East of England. (Since October 2019, this organisation has continued as the NIHR Applied Research Collaboration (ARC) East of England). The aims of the study were twofold: First, to identify the extent of PPI involvement in CLAHRC EoE funded studies; and second, to assess the usefulness of the GRIPP2 checklists to describe the reporting of PPI, from the perspective of lay co-researchers. The initial idea for the study was suggested by researchers in the NIHR CLAHRC EoE. They requested the lead researcher (JJ), who at the time, was new to her role at the University of Hertfordshire and had no existing collaboration with the NIHR CLAHRC EoE, to design and conduct a study to review the reporting of PPI in publications from the NIHR CLAHRC EoE funded studies.

## Methods

This is a descriptive study of all research publications published between 1st January 2014 and 31st October 2017, derived from research studies that were wholly or partially funded by the NIHR CLAHRC (EoE). This time period represents the start date of NIHR funding of the CLAHRC EoE (1st January 2014) and a cut-off date related to the scope of this study (31 October 2017). Research publications were identified from different sources, including: the CLAHRC EoE website publication list; CLAHRC EoE annual reports; information requested from researchers working within the CLAHRC EoE; and hand searching of identified publications for additional ‘companion’ articles from the same research projects. All the publications included in our review were published prior to the release of the GRIPP2 reporting checklists in 2017 [18, 30].

### Inclusion criteria

We included all research papers published during the defined time period that had some form of funding from the CLAHRC EoE. This included publications reporting fully funded CLAHRC projects, co-funded studies with another funder or organisation, and publications where one or more authors had received CLAHRC funding for their post during the time of their involvement with the reported research.

### Process

All publications identified were initially filtered by two reviewers (JJ a researcher and CP a research administrator), to establish eligibility in terms of funding from the CLAHRC EoE and to remove duplications from the different sources. The publications were then read by two assessors (researchers JJ and HW) to identify any mention of PPI activity. This involved checking all sections of the publications, including the main article, the abstract and keywords, acknowledgements section, and authors’ details and contributions.

### Involvement of lay co-researchers

In March 2017, a draft proposal for this study was discussed by the lead researcher (JJ) with members of the Public Involvement in Research group (PIRg), based at the University of Hertfordshire. The PIRg was established in 2005 and is composed of 14 members with lived experience of health conditions, experience of using health and social care services and/or caring for others. The PIRg members gave feedback on the design of the study and were asked for their interest to join the research team as co-researchers. Four PIRg members expressed interest in being involved.

In January 2018 the four co-researchers met with the lead researcher (JJ) to discuss the proposed review process using the GRIPP2 forms (Guidance for Reporting Involvement of Patients and the Public, version 2), which had recently been published [30]. Following a joint discussion regarding how best to approach the review process, the lead researcher (JJ) wrote some additional instructions written in plain language and developed ‘word versions’ of the GRIPP2 forms for the co-researchers to use for their review. This was because we could not find editable versions of the GRIPP2 forms on the EQUATOR website; we contacted the lead researcher (Professor Sophie Staniszweska) for permission to develop word versions of the short and long forms for use in this study. These word versions adapted for our study are shown as: Additional file 1 (short form); and Additional file 2 (long form). The co-researchers were provided with both forms (short and long) and used whichever form they considered most relevant for the publications they reviewed. The co-researchers reviewed in two pairs; each pair was given different types of studies and publications to review, in order to provide a range of studies to consider.

The four co-researchers reviewed eight publications each, between January and July 2018. The publications were reviewed over two time periods (January to March and May to July) to allow time for additional searching of eligible publications. Some co-researchers completed the GRIPP2 forms electronically, some hand wrote on the forms and posted to the lead researcher. Face to face discussions and telephone conversations were held between the lead researcher (JJ) and co-researchers between April–July 2018 to discuss the publications, the review process and their experiences of using the GRIPP2 forms (short and long). The lead researcher (JJ) wrote a first draft of this article, which was edited and revised by all co-authors. A GRIPP2 Long Form checklist for this article was completed by the lead researcher and is available as Additional file 3.

## Results

A total of 148 research publications published between 1st January 2014 and 31st October 2017 were identified, of which 114 met the inclusion criteria of being funded in some way by the CLAHRC EoE. Of the 34 excluded publications, most of them had one or more authors associated with CLAHRC EoE, but on closer scrutiny the research reported had not received any direct funding from the CLAHRC EoE (either for the project as a whole or a contribution to an author’s salary). Of the 114 publications, 16 (14%) reported some element of PPI activity and were included in the review.

Table 1 provides a summary of reported PPI activity in each publication, using the section headings of the GRIPP2-SF as a framework. To this table, we have added three main headings with additional aspects of PPI reporting considered important by the co-researchers, namely; whether or not there were PPI co-authors; whether PPI or similar terminology were included as key words or in the abstract (which is a section in the GRIPP2-LF, but not the GRIPP2-SF); and whether there was the mention of PPI contributors in the acknowledgement section.

**Table 1:**
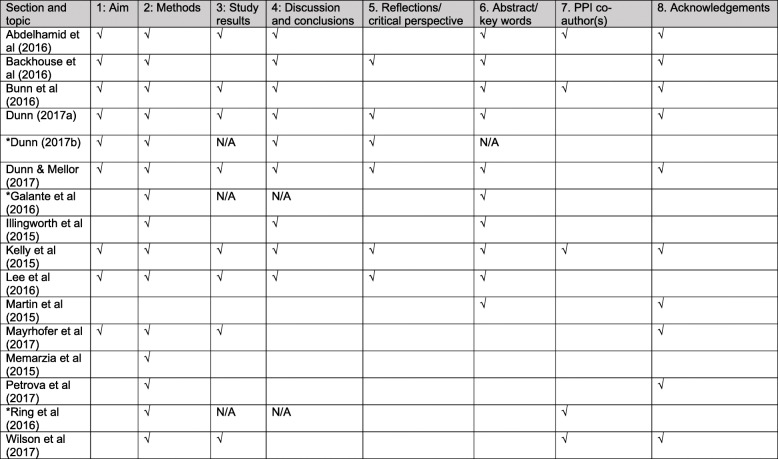
Studies reporting PPI in the publications using the GRIPP2-SF headings, with sections 6–8 added by the authors. (A tick represents when PPI was reported. N/A represents when a publication didn’t include a section heading, e.g. discussion and protocol publications *)

Table 2 describes the types of studies and publications reviewed. Two of the systematic reviews were ‘companion reviews’ [32, 33] from the same project. Four of the publications reported [34, 35], reflected on [36] and evaluated [37] the same programme of participatory research with young people, who were involved at different stages of the research process. In one publication, the authors used the GRIPP checklist (original 2011 version) to report the process and impact of PPI in their study [38].

**Table 2:**
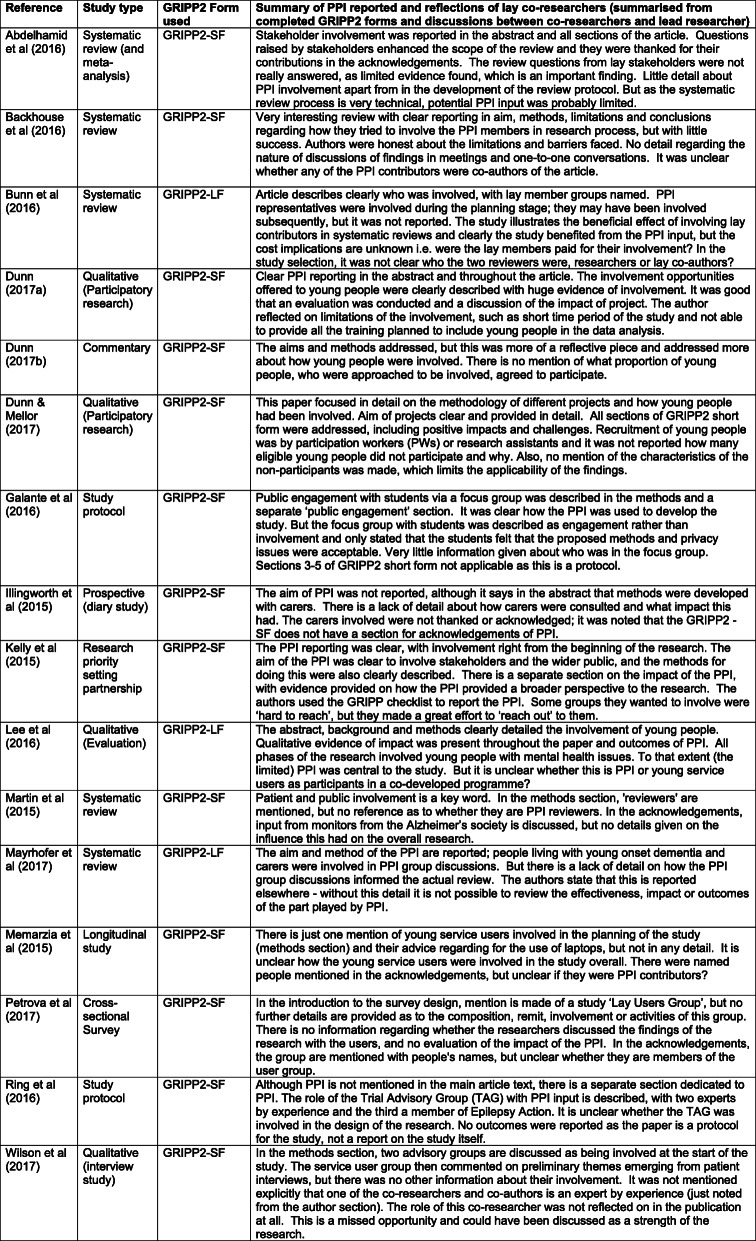
Type of study, GRIPP2 form used, summary and reflections from lay co-researchers

As shown in Table 2, for most of the publications the co-researchers chose to use the GRIPP2-SF. Table 2 also provides a summary of the PPI reported in the 16 publications, with the reflections on the quality of the PPI reporting from the perspectives of the four lay co-researchers. The summaries provided are based on the completed GRIPP2 forms and follow-up discussions between the lead researcher and the lay co-researchers. These summaries were written up in draft form by the lead researcher and then revised by the co-researchers. The perspectives of the co-researchers form the basis of this results section, with a discursive account of the strengths and limitations of the PPI reporting in the 16 publications, arranged by study type. This is followed by a summary of the co-researchers’ experiences and views of the GRIPP2 forms. When comments are made from a ‘co-researcher’, this refers to one of the four lay co-researchers in the research team.

### Systematic reviews

Five articles reported PPI in the systematic review process. The five systematic reviews were addressing review questions on older people, dementia and care homes. Two systematic reviews [32, 33] reported findings from the same study called the EDWINA (Eating and Drinking Well IN dementia) systematic review. The involvement of service users was reported in the abstracts and all sections of both articles. Lay stakeholders from four different service user organisations and groups were involved in the development of the review protocol and the identification of service user specific review questions, which were reported separately in the results sections. It was unclear whether the lay stakeholders were involved in subsequent stages of the reviews, although one co-researcher commented that the systematic review process is very technical, so potential input is probably limited. The impact of the PPI was reported differently in the two articles: in Abdelhamid et al. [32], it was stated that the scope of the review was enhanced by lay stakeholder input to ensure that relevant objectives were included; Bunn et al. [33] reported that the PPI was a strength of the review, but did not state what difference it made. All the lay stakeholders were acknowledged and staff representatives of AgeUK Norfolk and NorseCare were named as co-authors on both articles. In summary, one co-researcher commented on Bunn et al. [33] that “the study illustrates the beneficial effect of involving lay contributors in systematic reviews.”

Another systematic review focused on the involvement of older care-home residents as collaborators or advisors in research [39]. It clearly described how the research team attempted to involve PPI team members (care home residents, older people living in the community and care home staff) throughout the review process, but with limited success. PPI team members were involved in the planning of the review and discussing the findings, but barriers were encountered when trying to involve them in the review process itself. This was reflected upon honestly by the authors. However, there was little detail regarding the nature of discussions and PPI input in the meetings and conversations that were held. The PPI team members were acknowledged for their contributions in the acknowledgement section of the publication, but it was unclear whether any PPI members were co-authors. This was checked with the lead author by email, and it was confirmed that they were not named as co-authors.

Mayrhofer et al. [40] involved people living with young onset dementia and their carers in group discussions to inform the focus of this systematic review. In the acknowledgements, the Alzheimer’s Society were thanked for helping to facilitate the PPI discussions. In this publication, there was a lack of detail on how the PPI discussions influenced the review; it was stated that this would be reported elsewhere. A subsequent publication has since been published with considerable more detail of the PPI, but unfortunately this was published after this study had been completed [41].

In the systematic review by Martin et al. [42], the keyword ‘patient and public involvement’ and the mention of PPI representatives in acknowledgements section, both indicated PPI input in the research. In the acknowledgements, named representatives from the Alzheimer’s Society were thanked ‘for their support and input throughout the project’. However, no mention was found of any PPI activity in the main article, which was surprising as the acknowledgements suggested considerable PPI input in the project.

In summary, PPI has played a role in all the systematic reviews reported here. But there was great variation in the detail provided about the nature of the PPI activity, who was involved and the extent to which the PPI influenced the findings of the systematic reviews.

### Qualitative studies

Three of the publications reporting on qualitative research studies described different elements of a programme of participatory research with young people, involved at different stages of the research as co-researchers [34, 35, 37]. These publications reported in detail the young people’s involvement throughout the articles, including the abstracts. The PPI activities reported included the planning of projects, co-facilitating of focus groups, co-producing short films and involvement in dissemination, including conference presentations. However, the involvement in dissemination did not extend to being co-authors in these publications. In Dunn [34], the author reflected on limitations encountered around the PPI activities, including not being able to provide all the co-researchers with training on data analysis. This was due to limited time. The contributions of the young people, participation co-ordinators and partner organisations were acknowledged in the publications.

A mental health focused interview study [43] involved two PPI advisory groups (staff and mental health service users) at the planning and design stage of the research. The PPI groups provided feedback regarding recruitment, wording of information sheets, consent forms, and ethical considerations. Interview schedules were co-developed with the advisory groups and the service user group also commented on preliminary themes from patient interviews. However, it was considered a limitation that the role of a co-author, who was described as an ‘Expert by Experience’ was not reported, or reflected upon, as part of the research process. This was considered a missed opportunity, that could have been highlighted as a strength of the research overall.

### Quantitative studies

The quantitative studies provided a mixed picture of PPI reporting. A survey of General Practitioners (GPs) and Practice Managers [44] was designed with the input from a Lay User Group who were thanked for their contribution in the acknowledgements. However, no further details were provided as to the composition, remit, or further involvement of this group in the study. In a longitudinal study of young people leaving mental health or social care services [45] there was a single mention of PPI activity; young service users were mentioned as being involved in the planning of the study, and that their advice influenced the use of laptops to complete questionnaires. Individuals were mentioned in the acknowledgements, but it was unclear if they were PPI contributors. In a study led by Illingworth et al. [46], it was reported that 14 carers of adults with intellectual disability were involved in the development of diaries as a data collection method. However, there was no detail regarding how the carers were consulted and what impact this had on the research overall. A co-researcher also pointed out that the carers’ input was not acknowledged in the acknowledgements section.

Two study protocols for randomised controlled trials (RCTs) [47, 48] provided some examples of PPI input in the development of RCTs and future planning of the studies. In the protocol for the Mindful Student Study [47], it was clear to see how engagement with students in a ‘focus group’ (description used by authors) was used to inform the study design, specifically regarding data collection methods and privacy issues. However, very little information was provided regarding who was in the focus group, or how the students’ input influenced the study design. It just reported that the students felt that the proposed methods were ‘acceptable’. In the study protocol for a RCT to improve outcomes for adults with epilepsy and intellectual disability [48], the role of a Trial Advisory Group (TAG) was discussed. The advisory group included a carer of a person with severe intellectual difficulties (ID) and epilepsy, a home manager with experience of care for people with ID and epilepsy and a representative from the charity Epilepsy Action. Although it was unclear whether the TAG was involved in the study design, it was reported that the group’s advice was acted upon and reported throughout the trial. One of the co-authors was a staff representative from Epilepsy Action.

### Other types of publications

A publication reporting a Dementia research priority setting partnership (PSP) with the James Lind Alliance [38] clearly reported PPI in all sections of the article. The Alzheimer’s Society initiated the PSP with the University of Cambridge. The steering group included a PPI representative from the Alzheimer’s Society Research and a research officer from the Alzheimer’s Society was a co-author. The aim of the PPI was to involve people with dementia and their carers in the design, management and conduct of the project and the methods for doing so were clearly explained. There was a separate section on the impact of the PPI, with evidence on how the PPI provided a broader perspective to the research. Of significance, this was the only publication where the authors stated that they used the GRIPP checklist to report the process and impact of PPI.

Finally, a commentary paper by Valerie Dunn [36] provided a reflective account of involving young people in the creative participatory research projects already reported [34, 35, 37]. The aims and methods of PPI were addressed, but as commented by a co-researcher, this is more of a reflective piece about how the young people were involved.

### Lay co-researchers’ views of using the GRIPP2 long and short forms

As discussed in the methods, the four co-researchers were provided with both the GRIPP2 long and short forms; it was agreed that they would use whichever form (long or short) they considered most useful for the different publications they reviewed. In total, three publications were reviewed using the long form [33, 37, 40] and the other 13 were reviewed using the GRIPP2-SF. After both rounds of reviewing, the lay co-researchers were asked about their experiences of using the GRIPP2 forms by the lead researcher (JJ). After the first round, this involved one face to face meeting with three of the co-researchers and a telephone conversation with the fourth. After the second round of reviewing, individual telephone conversations were conducted with all four co-researchers.

The two co-researchers who used both the GRIPP2-SF and GRIPP2-LF, did so as they were interested to see how relevant and useful the different forms were for different types of studies. Following this exercise, they considered the short form most useful. They considered the long form to be inappropriate for the publications it was used for (two systematic reviews and an evaluation) as the issues addressed in the articles didn’t often conform to the GRIPP2 long form headings. The four co-researchers used phrases including ‘too complicated’, ‘needlessly repetitive’ and ‘a lot of duplication’ to describe the long form.. It was not considered to be ‘lay friendly’ and thought to be aimed at researchers rather than lay reviewers. The short form was viewed more positively as being ‘quite useful and straightforward’ and ‘particularly easy for a lay member’. It was also found to be a better format for different types of papers, although the structure didn’t always match the format of some types of articles. For example, the co-researchers found that using the GRIPP2-SF to review the two study protocols [47, 48] was limited, as due to the focus of a protocol, only the planning stage of the research process could be reported. However, one co-researcher said that using the GRIPP-SF for a non-empirical paper was useful as an aide-memoir and a useful framework ‘to remind you what you are looking for’.

It was considered that both the GRIPP2 forms would benefit from clearer instructions regarding how to use the forms, particularly for public reviewers. This became apparent when the co-researchers used the GRIPP2 forms differently, despite receiving the same training and guidance from the lead researcher (JJ). For example, some co-researchers noted the page numbers, others just provided their views overall. It was agreed that an important omission from both GRIPP2 forms was a separate section on whether PPI contributors had been acknowledged; in our study, the acknowledgement sections sometimes gave a clue that there had been some PPI in the research, even if not reported in the main sections of the article.

## Discussion

Our study found that 14% [5] of the publications, reporting research funded by the CLAHRC EoE (between 1 January 2014 and 31 October 2017), described some element of PPI activity. Some publications had clear and detailed reporting of PPI activities in many sections of the publications, including an account of the impact the PPI had made to the research. Whereas other publications showed limited PPI reporting and lacked detail in terms of describing who was involved, what they did and what difference it made. Due to the scope of our study, it remains unclear whether this was a result of minimal PPI activity in the studies reported, or suboptimal reporting of PPI in the publications. This could be a result of different contributing factors. As suggested by Price et al. [17], it may not simply be a case of poor PPI reporting, but the limited reporting requirements and word counts in some journals, which can play a part in ‘masking’ the actual PPI activity that took place. We recommend that journals have a PPI policy with clear guidance for authors to describe how members of the public were involved in the research being reported. Some journals have adopted this approach, such as the British Medical Journal (BMJ), the BMJ Open, Research Involvement and Engagement [26] and the British Journal of Occupational Therapy [49]. But this editorial practice is not consistent across health journals. Wider structural and organisational barriers must also be recognised, including a real-world reality that reporting on PPI may not always be a priority for some researchers, disciplines or journals [23, 50].

A previous study that explored the PPI in the NIHR CLAHRC EoE using a case study approach [51], has suggested that PPI was not fully embedded, active and comprehensive in all research studies within this CLAHRC. Our study has added to this picture, by identifying some examples of good practice in PPI and its reporting in publications, but also some suboptimal PPI reporting. We also identified significant ‘missed opportunities’ in some publications to describe PPI that had been undertaken, but that was not described in any detail. This reduced the potential for shared learning, and importantly, an understanding of how the involvement made a difference to the research [21]. An example is the systematic review by Martin and colleagues [42], which had ‘patient and public involvement’ as a key word and in the acknowledgement section, two named Alzheimer’s Society monitors (PPI contributors) were thanked ‘for their support and input throughout the project’. So it appears that there had been PPI input at different stages of the research; but this was not reported anywhere in the main paper and therefore the impact of the PPI remains ‘invisible’.

Another publication that was considered to be a ‘missed opportunity’ to report and reflect on the PPI was the mental health study led by Wilson and colleagues [43]. This was the only publication with a named co-author who was described as an ‘Expert by Experience’. We were surprised that there was no reflective commentary regarding the role played by this co-author, including how her personal lived experience of mental health care may have influenced the research. This, of course, may be because the co-author did not wish to divulge their personal experiences in a publication; but it was still considered a ‘missed opportunity’ by our co-researchers, that could have strengthened the account of the research from a PPI perspective. Particularly if the co-author had a co-researcher role in the research, which would have been a valuable experience to share with readers regarding the wearing of different ‘hats’ in research [52].

It is considered a benefit that our broad approach in this study provided the opportunity to evaluate different types of research studies using the GRIPP2 reporting checklists. This revealed innovative ways by which researchers, service users, carers and user organisations had collaborated in different types of research, including systematic reviews and study protocols. Three of the systematic reviews involved a wide variety of patients, carers and representatives from user-led organisations in different stages of the review process, using different methods of involvement, and reported this clearly in their publications, including in the abstracts [32, 33, 39]. One of our co-researchers commented that the public involvement discussed by Bunn et al. [33] really demonstrated the benefits of involving lay contributors in systematic reviews. The challenges of involvement in the systematic review process were also discussed openly by some authors; Backhouse and colleagues [39] provided an honest account of their largely unsuccessful attempts to involve care home residents in some stages of the systematic review process. These challenges were recognised by our co-researchers and also within the wider literature [53]. Although it remains the case that it is less common for researchers to report the challenges of PPI in research, or the wider systems within which it operates [54].

We found the GRIPP2-SF (short form) to be useful and straightforward, and generally appropriate for different types of studies, including protocols and systematic reviews. As commented by one co-researcher, the GRIPP2-SF was a useful framework ‘to remind you what you are looking for’. Whereas the GRIPP2-LF (long form) was considered by the co-researchers to be over complicated from a lay perspective. From the co-researchers’ use of the GRIPP2 checklists, they recommended having clear instructions for public reviewers regarding the reviewing process, focused on how to use the GRIPP2 reporting checklists and how to approach the reviewing process as a whole. Indeed, we consider that clearly written guidance is required for the critical appraisal of the whole of a journal article, not just the PPI aspects of the research. The importance of clear guidance and a user-friendly process for patient and public reviewers has also been highlighted by a survey of 227 patient and public reviewers of two health journals [55].

We are not aware of specific training and support for patient and public reviewers; guidance is offered by some journals, such as Research Involvement and Engagement, with peer review training resources signposted on the journal website for all peer reviewers. But these resources are not tailored specifically for patient and public reviewers, for whom peer reviewing may be a completely new experience. Indeed for some public reviewers, the experience of journal reviewing feels like they have been ‘dropped in at the deep end’ without adequate guidance, resources, support or reimbursement [56]. Guidance has been developed by the NIHR on training for public reviewers more generally, such as for reviewing research proposals, protocols, funding or grant applications [57]. But we are not aware of guidance for public reviewers for the peer reviewing of journal articles. We recommend the co-production of bespoke guidance and training for public reviewers of journal articles. This could be created and delivered together by members of the public, research organisations and journals, using a similar approach to the ‘lay assessor training’ which is part of a programme of shared resources called the Sharebank [56]. We also support the view that patient and public reviewers should be reimbursed for conducting journal reviews; expecting people to review for free is inequitable and places additional barriers to attracting diverse range of patient and public voices to journal reviewing [55].

To our knowledge, this is one of the first studies to explore PPI reporting with lay co-researchers, using the GRIPP2 reporting checklists. We are aware that the scoping review of dementia research by Miah and colleagues, published in 2019, used the GRIPP2-SF as a review framework and also involved lay members of a dementia research advisory group. But the advisory group members were consulted on the review findings, rather than being actively involved in the process of reviewing individual articles with the GRIPP2 checklists, as in our study.

Based on our experiences of the reviewing process from a lay co-researcher perspective, we recommend that some new sections are added to the GRIPP2-SF. A section to record whether PPI contributors are acknowledged for their involvement in the research is considered very important by our co-researchers. From our experience of undertaking this study, often the acknowledgement section provided an important clue regarding PPI input, which was particularly useful when there was either an absence, or limited detail of PPI activities reported in the main article. Two other suggested additions to the GRIPP2-SF are: to record whether PPI is reported in the abstract or key words (already a section in the long form); and a section to record whether there are PPI co-authors. These recommended additional sections are shown in Table 1.

Of significance to our study is that Miah and colleagues also adapted the GRIPP2-SF for their scoping review, adding five additional sections: country (where the study was conducted); PPI terms used; population (which patients or public population took part); evaluation methods (used to evaluate the impact of PPI); and findings from evaluation (the impact of the PPI on researchers, patients and public involved and on the research process). The final two items about capturing the impact of PPI are items from the GRIPP2-LF, but not currently in the GRIPP2-SF. As discussed by the authors, this is a current limitation of the GRIPP2-SF, which has a particular emphasis on identifying and describing PPI activities, but not evaluating the quality and impact of the PPI. We agree that a future version of the GRIPP2-SF could consider incorporating items on PPI impact, in addition to other recommendations made by the co-researchers from our study. As with the development of the GRIPP2 reporting checklists, a revised version should be developed in collaboration with patients and members of the public, to incorporate their experiences of reviewing, including using the GRIPP2 and other relevant frameworks. We also acknowledge that there are different approaches to reporting and evaluating PPI in research, with PPI considered as an essential part of the research methodology. This can include reflective approaches, with personal accounts from researchers and members of the public on how involvement worked (and when it didn’t, or was difficult) and the shared learning gained [21].

The involvement of four co-researchers as members of the research team is considered a great strength of this study. This enabled an exploration of the usefulness of the GRIPP2 checklists from the perspectives of patient and public reviewers. Another strength of our study is that we included a breadth of study designs and publication types in the review, published in a range of health and social care journals. This has demonstrated the usefulness of the GRIPP2 reporting checklists, particularly the short form (GRIPP2-SF), to review PPI reporting in different types of research studies and publications. We acknowledge several limitations to our study. Due to the scope of our research, we only included research publications funded by one organisation, the CLAHRC EoE. Great efforts were made to identify all research papers published during the study time period, but we may have missed some eligible publications. Although most of the publications were reviewed using the GRIPP2-SF, three publications were reviewed using the GRIPP2-LF and so there may be some inconsistency in the reviewing process. We also acknowledge that due to the timing and scope of this study, the publications reviewed were published before the publication of the GRIPP2 checklists [18, 30]. Finally, we recognise that with the exception of our study and the review by Miah and colleagues [2], there remains limited evidence regarding how the GRIPP2 reporting checklists are being used, by whom and to what effect. But over time, we anticipate further publications that share experiences and evidence of the strengths and limitations of the GRIPP2 reporting checklists. This will enhance our knowledge of its application and contribute to the growing evidence base of PPI.

## Conclusions

To our knowledge, this is one of the first studies to include lay co-researchers using the GRIPP2 reporting checklists (GRIPP2-SF and GRIPP2-LF) to peer review different types of research publications. Out of 148 research publications identified, 16 (14%) reported some aspect of PPI activity. When using the GRIPP2 checklists as a framework, we found great variation in the frequency and quality of PPI reporting in the 16 publications we reviewed. Some research studies were exemplars in PPI reporting, with a detailed account of PPI activities in the different stages of the research process. Other publications showed limited PPI reporting, with some examples of what was perceived as ‘missed opportunities’ to showcase PPI in the research being presented. We are mindful that journals have different word limits and requirements for PPI reporting, and this may contribute to the variations observed. We recommend that journal editors re-consider overly restrictive rules on word lengths and provide authors with the opportunity, with editorial guidance, to clearly describe how members of the public were involved in the research being reported. There are now some excellent examples of PPI policies and statements in health journals, developed in collaboration with patients and members of the public [26, 49], demonstrating a recognition by journal editors of the value and impact of PPI in research. It is recommended that user-friendly PPI reporting checklists, such as the GRIPP2, continue to be used by lay reviewers and researchers alike, to ensure that clear and open reporting of PPI, addressing the successes and failures of PPI, are documented in research publications. Widespread and accessible training and support for patient and public reviewers, co-created with members of the public,, is also recommended. These welcome developments will enhance our shared learning and understanding of what meaningful PPI looks like in publications.

## Supplementary Information

**Additional file 1.** GRIPP2 short form.

**Additional file 2.** GRIPP2 long form.

**Additional file 3.** Completed GRIPP2 long form.

## Data Availability

Not applicable.

## Abbreviations

GRIPP2: Guidance for Reporting Involvement of Patients and the Public (Version 2)
PIRG: Public Involvement in Research Group
RAPPORT: ReseArch with Patient and Public invOlvement: a RealisT evaluation
