# Supplementary material for: Reporting on patient and public involvement (PPI) in research publications: using the GRIPP2 checklists with lay co-researchers
Source: Res Involv Engagem. 2021 Jul 22;7:52. doi: 10.1186/s40900-021-00295-w (PMC8296743; doi:10.1186/s40900-021-00295-w)
Supplement: Supplementary file 1 — Additional file 1. GRIPP2 short form. [file 40900_2021_295_MOESM1_ESM.pdf]

GRIPP2 short form

Section and topic	Item	Reported on page No
1: Aim	Report the aim of PPI in the study	
2: Methods	Provide a clear description of the methods used for PPI in the study	
3: Study results	Outcomes—Report the results of PPI in the study, including both positive and negative outcomes	
4: Discussion and conclusions	Outcomes—Comment on the extent to which PPI influenced the study overall. Describe positive and negative effects	
5: Reflections/critical perspective	Comment critically on the study, reflecting on the things that went well and those that did not, so others can learn from this experience	

PPI=patient and public involvement
