# Supplementary material for: Reporting on patient and public involvement (PPI) in research publications: using the GRIPP2 checklists with lay co-researchers
Source: Res Involv Engagem. 2021 Jul 22;7:52. doi: 10.1186/s40900-021-00295-w (PMC8296743; doi:10.1186/s40900-021-00295-w)
Supplement: Supplementary file 2 — Additional file 2. GRIPP2 long form. [file 40900_2021_295_MOESM2_ESM.pdf]

GRIPP2 long form

Section and topic	Item	Reported on page No
Section 1: Abstract of paper		
1a: Aim	Report the aim of the study	
1b: Methods	Describe the methods used by which patients and the public were involved	
1c: Results	Report the impacts and outcomes of PPI in the study	
1d:Conclusions	Summarise the main conclusions of the study	
1e: Keywords	Include PPI, “patient and public involvement,” or alternative terms as keywords	
Section 2: Background to paper		
2a: Definition	Report the definition of PPI used in the study and how it links to comparable studies	
2b: Theoretical underpinnings	Report the theoretical rationale and any theoretical influences relating to PPI in the study	
2c: Concepts and theory development	Report any conceptual models or influences used in the study	
Section 3: Aims of paper		
3: Aim	Report the aim of the study	
Section 4: Methods of paper		
4a: Design	Provide a clear description of methods by which patients and the public were involved	
4b: People involved	Provide a description of patients, carers, and the public involved with the PPI activity in the study	

Section and topic	Item	Reported on page No
4c: Stages of involvement	Report on how PPI is used at different stages of the study	
4d: Level or nature of involvement	Report the level or nature of PPI used at various stages of the study	
Section 5: Capture or measurement of PPI impact		
5a: Qualitative evidence of impact	If applicable, report the methods used to qualitatively explore the impact of PPI in the study	
5b: Quantitative evidence of impact	If applicable, report the methods used to quantitatively measure or assess the impact of PPI	
5c: Robustness of measure	If applicable, report the rigour of the method used to capture or measure the impact of PPI	
Section 6: Economic assessment		
6: Economic assessment	If applicable, report the method used for an economic assessment of PPI	
Section 7: Study results		
7a: Outcomes of PPI	Report the results of PPI in the study, including both positive and negative outcomes	
7b: Impacts of PPI	Report the positive and negative impacts that PPI has had on the research, the individuals involved (including patients and researchers), and wider impacts	
7c: Context of PPI	Report the influence of any contextual factors that enabled or hindered the process or impact of PPI	
7d: Process of PPI	Report the influence of any process factors, that enabled or hindered the impact of PPI	

Section and topic	Item	Reported on page No
7ei: Theory development	Report any conceptual or theoretical development in PPI that have emerged	
7eii: Theory development	Report evaluation of theoretical models, if any	
7f: Measurement	If applicable, report all aspects of instrument development and testing (eg, validity, reliability, feasibility, acceptability, responsiveness, interpretability, appropriateness, precision)	
7g: Economic assessment	Report any information on the costs or benefit of PPI	
Section 8: Discussion and conclusions		
8a: Outcomes	Comment on how PPI influenced the study overall. Describe positive and negative effects	
8b: Impacts	Comment on the different impacts of PPI identified in this study and how they contribute to new knowledge	
8c: Definition	Comment on the definition of PPI used (reported in the Background section) and whether or not you would suggest any changes	
8d: Theoretical underpinnings	Comment on any way your study adds to the theoretical development of PPI	
8e: Context	Comment on how context factors influenced PPI in the study	
8f: Process	Comment on how process factors influenced PPI in the study	
8g: Measurement and capture of PPI impact	If applicable, comment on how well PPI impact was evaluated or measured in the study	

Section and topic	Item	Reported on page No
8h: Economic assessment	If applicable, discuss any aspects of the economic cost or benefit of PPI, particularly any suggestions for future economic modelling.	
8i: Reflections/critical perspective	Comment critically on the study, reflecting on the things that went well and those that did not, so that others can learn from this study	

PPI=patient and public involvement
