# Supplementary material for: Reporting on patient and public involvement (PPI) in research publications: using the GRIPP2 checklists with lay co-researchers
Source: Res Involv Engagem. 2021 Jul 22;7:52. doi: 10.1186/s40900-021-00295-w (PMC8296743; doi:10.1186/s40900-021-00295-w)
Supplement: Supplementary file 3 — Additional file 3. Completed GRIPP2 long form. [file 40900_2021_295_MOESM3_ESM.pdf]

GRIPP2 long form

Section and topic	Item	Reported on page No
Section 1: Abstract of paper		
1a: Aim	Report the aim of the study	P2
1b: Methods	Describe the methods used by which patients and the public were involved	P2
1c: Results	Report the impacts and outcomes of PPI in the study	P2
1d:Conclusions	Summarise the main conclusions of the study	P3
1e: Keywords	Include PPI, "patient and public involvement," or alternative terms as keywords	P3
Section 2: Background to paper		
2a: Definition	Report the definition of PPI used in the study and how it links to comparable studies	P4
2b: Theoretical underpinnings	Report the theoretical rationale and any theoretical influences relating to PPI in the study	
2c: Concepts and theory development	Report any conceptual models or influences used in the study	P4-5
Section 3: Aims of paper		
3: Aim	Report the aim of the study	P7
Section 4: Methods of paper		
4a: Design	Provide a clear description of methods by which patients and the public were involved	P8-10
4b: People involved	Provide a description of patients, carers, and the public involved with the PPI activity in the study	P8-9

Section and topic	Item	Reported on page No
4c: Stages of involvement	Report on how PPI is used at different stages of the study	P8-10
4d: Level or nature of involvement	Report the level or nature of PPI used at various stages of the study	P8-10
Section 5: Capture or measurement of PPI impact		
5a: Qualitative evidence of impact	If applicable, report the methods used to qualitatively explore the impact of PPI in the study	P17-19
5b: Quantitative evidence of impact	If applicable, report the methods used to quantitatively measure or assess the impact of PPI	
5c: Robustness of measure	If applicable, report the rigour of the method used to capture or measure the impact of PPI	
Section 6: Economic assessment		
6: Economic assessment	If applicable, report the method used for an economic assessment of PPI	
Section 7: Study results		
7a: Outcomes of PPI	Report the results of PPI in the study, including both positive and negative outcomes	P17-19, p21-23
7b: Impacts of PPI	Report the positive and negative impacts that PPI has had on the research, the individuals involved (including patients and researchers), and wider impacts	P21-23
7c: Context of PPI	Report the influence of any contextual factors that enabled or hindered the process or impact of PPI	P8-9
7d: Process of PPI	Report the influence of any process factors, that enabled or hindered the impact of PPI	P8-9

Section and topic	Item	Reported on page No
7ei: Theory development	Report any conceptual or theoretical development in PPI that have emerged	
7eii: Theory development	Report evaluation of theoretical models, if any	
7f: Measurement	If applicable, report all aspects of instrument development and testing (eg, validity, reliability, feasibility, acceptability, responsiveness, interpretability, appropriateness, precision)	
7g: Economic assessment	Report any information on the costs or benefit of PPI	
Section 8: Discussion and conclusions		
8a: Outcomes	Comment on how PPI influenced the study overall. Describe positive and negative effects	P23
8b: Impacts	Comment on the different impacts of PPI identified in this study and how they contribute to new knowledge	P23
8c: Definition	Comment on the definition of PPI used (reported in the Background section) and whether or not you would suggest any changes	
8d: Theoretical underpinnings	Comment on any way your study adds to the theoretical development of PPI	
8e: Context	Comment on how context factors influenced PPI in the study	
8f: Process	Comment on how process factors influenced PPI in the study	
8g: Measurement and capture of PPI impact	If applicable, comment on how well PPI impact was evaluated or measured in the study	P23

Section and topic	Item	Reported on page No
8h: Economic assessment	If applicable, discuss any aspects of the economic cost or benefit of PPI, particularly any suggestions for future economic modelling.	
8i: Reflections/critical perspective	Comment critically on the study, reflecting on the things that went well and those that did not, so that others can learn from this study	P22-23

PPI=patient and public involvement
